# Superinfection interference of alpha and orthoflavivirus pathogens by homologous non-pathogenic vaccine strains

**DOI:** 10.1128/spectrum.03362-24

**Published:** 2025-11-03

**Authors:** Julie Hicks, Dennis T. Brown, Richard A. Anthony, Hsiao-Ching Liu, Raquel Hernandez

**Affiliations:** 1 Department of Animal Science, NC State University 6798 , Raleigh, North Carolina, USA; 2 Department of Molecular and Structural Biochemistry, NC State University 6798 , Raleigh, North Carolina, USA; 3 Vacunax Inc, Camas, Washington, USA; Emory University School of Medicine, Atlanta, Georgia, USA

**Keywords:** arbovirus, superinfection exclusion, virus spread, co-infections

## Abstract

We have developed host-restricted, live attenuated virus (HR-LAV) vaccine strains of six tropical disease pathogens (chikungunya and dengue 1–4) that are non-pathogenic in vertebrates. The HR-LAV vaccine strains replicate efficiently in insect cells but are severely impaired in their replication within vertebrate cells, and the large deletions in these vaccine strains effectively prevent reversion to the wild type (WT) in the restrictive host. Here, we describe a novel approach that uses these homologous, but non-pathogenic HR-LAV vaccine strains to disrupt replication of secondary infections of WT pathogenic virus through the mechanism of Superinfection Exclusion, an intrinsic insect mechanism of virus replication control. Our results demonstrate that persistent primary infections of HR-LAV strains show a significant degree of pathogen interference in several mosquito cell lines, which may also be used to limit replication of WT virus in the whole mosquito.

**IMPORTANCE:**

We describe experiments that suggest a theoretical approach for mitigating the spread of mosquito-vectored diseases of medical importance. This method would exploit the phenomenon of superinfection exclusion, in which the presence of a virus in a cell prevents the expression of a second infecting virus. These data suggest that further studies in live insects are merited. Such studies may reveal that this method may be employed to replace the current mosquito control technologies that are environmentally unfriendly and harmful to humans, animals, and other insects that are important to the ecosystem.

## INTRODUCTION

Mosquitoes are the world’s most predominant carriers of insect-vectored human pathogens. They have been referred to as “the world’s deadliest animal” (https://www.cdc.gov/globalhealth/stories/2019/world-deadliest-animal.html). Of the >3,500 mosquito species, only a few transmit disease (1, 2). *Aedes aegypti* is the most common and versatile vector of virus pathogens because of its genetic predisposition to adapt to urban and rural environments, diurnal behavior, predilection to feed on humans, and the ability to transmit a variety of virus pathogens (3). It is estimated that 80% of the world’s population lives in areas supporting infestations by this mosquito in environments poised to harbor the most serious of these pathogens 4(5, 6). These include dengue virus (DENV), chikungunya virus (CHIKV), Zika virus (ZIKV) (7), and yellow fever virus, which cause febrile, arthritogenic, neuroinvasive, and hemolytic diseases that are of global medical and economic importance (8, 9). Dengue virus circulates in areas in which half of the world’s population is exposed (10) and is becoming an increasing threat in the US. The ability to infect both mosquitoes and vertebrates enables maintenance of arboviruses in nature in enzootic cycles with spillover into the human population. A second pervasive vector, the highly invasive *Aedes albopictus* mosquito (11), has been found to be the primary vector of CHIKV and ZIKV (12), making these two *Ae. aegypti* and *Ae. albopictus* the major vector of arbovirus disease (13–15). What were once tropical diseases restricted to the warmest regions of the planet, climate change has permitted their insect vectors to spread to their geographic range north, transporting these pathogens along with them (16). In spite of over 50 years of intensive effort to develop prophylactic methods to treat these illnesses, no treatment or globally accepted vaccines exist (17, 18).

Mosquito population control (19) has been and continues to be the most effective method to minimize the spread of the vectors and concomitant arbovirus diseases (20, 21) but has had significant ecological drawbacks (22).

To date, the most advanced biological mosquito control methods involve genetic or mechanical manipulation of the mosquito population. The most effective methods interfere with mosquito fertility and reproduction to inhibit pathogen transmission. Male mosquitoes sterilized through irradiation or genetic modifications have been used to reduce mosquito populations in the wild. This is the sterile insect technique and requires large and repeated release of mosquitoes to reduce the mosquito population (23). Unfortunately, lowering the mosquito populations in their respective environs affects a primary food source for the beneficial fauna of the ecosystem. A recent, more effective bio-control approach involves infecting mosquitoes with *Wolbachia*, an arthropod Rickettsiales endosymbiont (12). There are two main hypotheses to explain *Wolbachia* interference in viral replication: the activation of vector-host immunity and competition with the virus for cellular resources (24–26). Regardless of the mechanism of arbovirus bio-interference, *Wolbachia* treatment was shown to affect up to 98% of the mosquito population in one study (27). However, *Wolbachia* is not usually found in the *A. aegypti* mosquito, so a modified form must be established in the laboratory before releasing infected mosquitoes into the wild, and then this requires sustained maintenance release at a substantial cost as a biocontrol method (28, 29). Considering the limitations of the existing mosquito control strategies, it becomes clear that novel arbovirus mitigation methods are necessary.

Nasar et al. have demonstrated that the mosquito-specific alpha virus Eilat virus can produce homologous and heterologous interference of other alphaviruses *in vitro* and *in vivo*. In this study, we wish to extend these results to alpha- and flaviviruses of medical significance. Here, we provide evidence that an alternative biological method of mitigating circulating arbovirus pathogens in mosquito populations may be possible through the application of genetically modified, non-pathogenic forms of specific virulent arboviruses to induce targeted superinfection exclusion (SIE) (30–32). Viral SIE is defined by an initial primary infection preventing the replication of a secondary infection by a genetically identical or closely related virus. This phenomenon manifests as homologous interference or heterologous interference, depending on whether the interference occurs between a homologous or heterologous virus, respectively. This approach to mitigate pathogenic virus spread has been suggested previously, but a means of implementation had not been elaborated (32–34). To circumvent the risk of disease, we propose to establish SIE through a primary exposure of our non-pathogenic, host-restricted, live attenuated virus (HR-LAV) strains, developed as vaccines, as a means to restrict the growth of a secondary superinfection of a target pathogen, essentially vaccinating the mosquito host against the challenge virus. Our HR-LAV strains have been previously described and include mosquito-restricted host range deletion mutants for DENV 1-4, CHIKV, and Sindbis virus (SINV) (35–37). These HR-LAV strains for DENV 1–4, CHIKV, and SINV have been constructed and evaluated as vaccines for safety and protective efficacy in appropriate animal models, including non-human primates (35–37). Attenuation of the wild-type (WT) viruses HR-LAV constructs uses a common platform that deletes large segments of the structural glycoproteins’ transmembrane domain (TMD) (38) of the proteins that interact with the virus capsid protein, such as E (orthoflaviviruses) (39) or E2 (alphaviruses). We have found that deleting specific sequence motifs produces insect (host)-restricted LAV. These sequence motifs have been found to be necessary for the replication of the viruses in the vertebrate hosts but are nonessential to function in the mosquito membrane. Once the disruptive motif is identified, the same or similar motif is deleted in genetically related viruses, making HR-LAV construction a streamlined process that can theoretically be applied to any membrane-containing arbovirus. These are large TMD deletions—four amino acid residues in the orthoflaviviruses and nine amino acid deletions in the alphaviruses—are permanent, and no reversion to wild-type virus has been documented. For example, in the orthoflaviviruses, these deletions were not found to revert after six passages in vertebrate cell culture or after 6 days of infection in vervet monkeys (36). The mosquito-restricted HR phenotype has been attributed to the unique physiology and biochemistry of the mosquito membrane (38, 40). Distinct from mammals, the mosquito membrane is a structure of reduced vertical dimensions containing shorter lipids, no native cholesterol, and is biochemically disparate from mammalian membranes (41).

## MATERIALS AND METHODS

### Cells and viruses

Five mosquito cell lines were used in this study: three *Ae. albopictus* lines, C6/36, C7-10, and U4.4 (42), and two *A. aegypti* lines, Aag2 (obtained from Ana Sesma, Icahn School of Medicine at Mount Sinai (ISMMS) and A20 (obtained from Zachary Adelman, Texas A&M University). For the DENV 1–4 and CHIKV experiments, all cells were cultured in minimal essential medium (MEM) (Earle’s salts; Thermo Scientific) supplemented with 10% fetal bovine serum (FBS; Atlanta Biologicals), 2 mM glutamine (Thermo Scientific), and 10% tryptose phosphate broth (BD), at 28°C with 5% CO_2_. The dengue HR-LAVs (DV1 ILLT, DV2 GVII, DV3 GVLL, and DV4 GFLV) and WT (DENV1 WP74, DENV2 16881, DENV3 CH53489/UNC3001, and DENV4 341750) strains used in this study have been described previously (37). CHIKV 181/25 was obtained from BEI Resources. The CHIKV TM17 HR-LAV was described previously (35). For SINV experiments, all cells were cultured in MEM (Hanks salts; without sodium bicarbonate; Sigma) supplemented with 10% fetal bovine serum (Atlanta Biologicals), 2 mM glutamine (Thermo Scientific), and 10% tryptose phosphate broth (BD), at 28°C without CO_2_. The SINV TM17 HR-LAV and Sindbis virus heat-resistant (SVHR) WT strain have been described previously (38, 43).

### Establishment of infected cell lines and SIE experiments

Cells were infected at a multiplicity of infection (MOI) = 0.01 with the appropriate HR-LAV, infections were maintained, and cells were passaged as needed for at least a month. Infections were monitored weekly via conventional plaque assay. Cells were considered infected once virus concentrations leveled off from acute infection levels and titers remained consistent (44).

For the SIE experiments, cells (either infected with an HR-LAV or non-PI cells) were seeded at a density of 1 × 10^6^ in 24-well plates (Corning) in triplicate in serum-free media and allowed to adhere at 28°C for 1 h. Cells were then infected with the appropriate WT strain at MOI = 10 and incubated at room temperature (RT) with gentle rocking for 1 h. The inoculum was removed, cells were washed with 1× phophate buffered saline (PBS), and fresh growth medium was added. Culture media were collected and replaced with fresh media at 24 h, 48 h, and 72 h post-secondary infection (45) and centrifuged at 1,000 rpm for 10 min to remove any cellular debris. Glycerol was added at a final concentration of 10%, and samples were snap-frozen in liquid nitrogen and stored at −80°C until analysis.

### Fluorescent *in situ* hybridization determination of virus titers

In order to differentiate between the HR-LAVs and their WT counterparts, fluorescent *in situ* hybridization (FISH) (46) was employed using biotinylated probes designed to span the genomic deletion site of the HR-LAVs. Probe sequences are as follows: DV1 ILLT-5ʹ-GTTTAATCCTAGCCACCCTATTCCTATCTTC-3ʹ; DV1 WP74-5ʹ-TCCTAGCCATGTCAGCAGAATCCCTATTCC-3ʹ; DV2 GVII-5ʹ-ATTCCTATCCATGTTATGAGGATTTTCATA-3ʹ; DV2 16681-5ʹ-TCCATGTGATAATGACTCCTATGAGGATTT-3ʹ; DV3 GVLL-5ʹ-CAACCCTATCCAAGTTATTCCAATTTTCAT-3ʹ; DV3 CH-UNC-5ʹ-CCCTATCCAAGTCAAGAGAACAACTATTCC-3ʹ; DV4 GFLV-5ʹ-CGTGCCAATCCACAAAATTAGGATTCTAAT-3ʹ; DV4 341750-5ʹ-CCACAACACTAAGAACCCAATTAGGATTCT-3ʹ; CHIKV TM17-5ʹ-TGTGCCCACCGACACAATGACTACAGTCAT-3ʹ; CHIKV 181/25-5ʹ-CACACCCACCATCGACAGGAGTACGAACGA-3ʹ; SINV TM17-5ʹ-TAACACTGCAACAGTTACGCCGACGGCTAA-3ʹ; SVHR-5ʹ-CAATCATCATCGCCACGGTAGCTGATGCGA-3ʹ. All probes were 5ʹ-biotin conjugated and HPLC purified (Sigma). Probes were confirmed to be strain specific, i.e., the probes for the HR-LAVs did not bind to their WT counterpart, and *vice versa*.

C6/36 cells were seeded at 2 × 10^5^ cells per well in 96-well plates in serum-free media and allowed to adhere at 28°C for 1 h. For each SIE sample, 10-fold serial dilutions were made in 1× Hanks Buffered Salt Solution (Corning) containing 3% FBS. Cells were infected in triplicate for each dilution and incubated at room temperature for 1 h. The inoculum was then removed, and fresh growth medium was added. For the dengue SIE samples, cells were fixed 4 days post-infection, and for the CHIK and SINV SIE samples, cells were fixed 2 days post-infection. Cells were washed twice in 1× PBS and then fixed in 1× PBS containing 3.7% formaldehyde (Sigma) at RT for 20 min. Cells were washed twice with 1× PBS and then incubated in permeabilization buffer (1× PBS and 0.1% Triton X-100 [Sigma]) at room temperature for 20 min. Cells were washed twice with 1× PBS and twice with hybridization buffer (10% formamide [EMD Millipore], 2× SSC [Thermo Scientific], 0.1% Tween-20 [Sigma], 2 mM vanadyl ribonucleoside complex [VRC; New England Biolabs], 250 ng/µL sonicated/sheared salmon sperm DNA [Takara Bio], 250 ng/µL C6/36 Cot DNA, and 10% dextran sulfate [Sigma]). Cells were incubated in hybridization buffer at 37°C for 2 h. Plates were heated at 80°C for 10 min (using a slide warmer). Probes were denatured at 100°C for 5 min and then quickly added at a final concentration of 5 µM. Plates were sealed using aluminum foil sealing film and incubated at 37°C for 24 h. Cells were consecutively treated with: (i) one wash: 2× SSC, (ii) one wash: 1× SSC, and (iii) three washes: 1× PBS 0.1% Tween-20. All washes were incubated at 37°C for 5 min. Blocking buffer (2× SSC; 0.1% Tween-20; 1% BSA [heat-shock fraction; Sigma]; 1% egg white; 2 mM VRC) was heated at 37°C and added to each well, and plates were incubated at 37°C for 1 h. The buffer was removed, and a blocking buffer containing 5 µg/mL of Fluorescein (DTAF) Streptavidin (Jackson ImmunoResearch), heated at 37°C, was added. Plates were incubated (protected from light) at 37°C for 2 h. Cells were washed three times in 1× PBS containing 0.1% Tween-20 for 5 min each wash. Cells were counterstained with Hoechst 33342 (MP Biomedicals) and washed once with 1× PBS. The number of fluorescent foci in each well was determined using a Leica DMIL fluorescent microscope and then used to calculate virus titers. All statistical analyses and graphs were produced using GraphPad Prism.

C6/36 Cot DNA was generated as follows. One confluent T75 flask of C6/36 cells was washed three times with 1× PBS, then 10 mL of 1× TE (pH 8.0) was added, and a cell scraper was used to detach cells. Cells were centrifuged for 10 min at 1,000 rpm, 10°C. The cell pellet was resuspended in 3 mL of cell lysis buffer (10 mM Tris-Cl [pH 8.0], 0.1 M EDTA [pH 8.0], 0.5% SDS, 20 µg/mL DNase-free pancreatic RNase, and 100 µg/mL Proteinase K) and incubated at 50°C 3 h. Lysate was cooled to room temperature, and an equal volume of phenol (equilibrated with 0.1M Tris-Cl, pH 8.0; Sigma) was added and mixed on a tube rotator for 10 min. Lysate was centrifuged at 6,500 rpm for 15 min at 22°C. The aqueous phase was transferred to a new tube. The phenol extraction was repeated, and the aqueous phases were pooled. Then an equal volume of phenol:chloroform:isoamyl alcohol (IAA; 25:24:1; Sigma) was added to the aqueous phase, and the tube was gently inverted and centrifuged at 6,500 rpm for 15 min at 22°C. The aqueous phase was transferred to a new tube, and 0.2 vol of 10M ammonium acetate and 2.5 vol of 100% EtOH were added and mixed and left at RT until the DNA precipitant formed (~5 min). DNA was centrifuged for 5 min at 6,500 rpm at 22°C. The pellet was washed twice with 70% EtOH and air-dried. DNA was dissolved in 50–100 µL (depending on the size of the pellet) of 1× TE (pH 8.0) for 24 h at 4°C. Electrophoresis was used to confirm the extraction of high molecular weight DNA. The C6/36 genomic DNA was diluted to 10 µg/µL in 1.2× SSC. For each Cot fraction, 500 µL of DNA was boiled for 2 min then placed in a preheated 60°C heat block. For the Cot2 fraction, the DNA was removed after 1 min of incubation and placed on ice for 2 min. For the Cot3 fraction, the DNA was removed after 1.5 min of incubation and placed on ice for 2 min. To each fraction, 55 µL of preheated (42°C) 10× S1 nuclease buffer and 5 µL S1 nuclease (100 U/µL; Promega) were added, and the reaction was incubated at 42°C for 1 h. Then 0.1 vol of 3M sodium acetate (pH 5.2) and 1 vol of isopropanol were added and mixed and left at room temperature until the DNA precipitant formed (~5 min). DNA was centrifuged at 14,000 rpm for 20 min at 4°C. The pellet was washed twice with 70% EtOH, air-dried, and dissolved in 100 µL of water. Confirmation of fractionation was done using electrophoresis. Equal concentrations of the Cot2 and Cot3 fractions were pooled for use in the FISH buffer.

## RESULTS

Insect cell culture is a simple method of evaluating virus infection of a whole mosquito because many features of the virus replication are indistinguishable between *in vitro* and *in vivo* infection. As our goal is to manipulate the SIE phenomenon *in vivo*, whole mosquitoes, we began by examining the SIE effects of our HR-LAV strains that were infected in three cell lines of *Ae. albopictus* (U4.4 C7-10 and C6/36), and two cell lines of *Ae. aegypti* (Aag2 and A20), from the two most relevant mosquito vectors for DENV1-4 and CHIKV, and then challenge these with their pathogenic homolog. We present these data here and make the argument that the preliminary data *in vitro* is predictive of a robust SIE response *in vivo*. We examined this phenomenon in cell lines from different mosquito species, cell lifecycle, and tissue lineages because it is thought that each culture may display different infection and physical profiles that may impact the SIE phenomenon (47). Of the arbovirus group, SINV, the prototypical Alphavirus, is the best studied virus system for which the mechanism by which SIE has been elucidated (48–50). Therefore, SINV serves as the WT control and reference model in these studies.

In the literature, previous data reporting SIE in alphavirus and orthoflaviviruses, whether *in vitro* and *in vivo*, are incomplete, and the methods used to measure interference are inconsistent, difficult to interpret, or reproduce (32, 33, 51, 52 reviewed in [42]). Nonetheless, several consistent features of the superinfection interference phenomenon are known for the alpha and orthoflavivirus genera and have been applied to our studies. In the alphavirus system, SIE is established within 15 min of infection (48). Briefly, in SINV, SIE is the result of the RNA polymerase auto-processing into non-structural replicase complexes that can only produce +RNA. Replication of the genomic RNA is fast, and SIE is established early during the infection. Any incoming homologous virus +RNA cannot be transcribed into the negative strands required for a second homologous virus to be replicated because the necessary replicase is no longer available. In our proposed biocontrol approach in mosquitoes in the wild, SIE would be established in HR-LAV-infected mosquitoes. Thus, upon a primary infection of HR-LAV, we cultivated infected cells, allowing the primary infection to proceed until firmly established for 30 days. The 30-day period of infection allows the cells to proceed from the acute, lytic phase of infection into the persistent state (44).

It was also necessary to establish the MOI required for >95% of the cells to be infected to produce a single cycle of infection for the second challenge infection to provide an accurate assessment of interference. This ensures that the secondary infection is uniformly infecting all the cells to correctly quantify the virus load during the SIE period analyzed. The effective MOI determined by immunofluorescence was MOI = 10 pfu/mL for orthoflavivirus and ~1 pfu/mL for alphaviruses. The establishment of SIE in orthoflavivirus is slower than that of the alphavirus system, with interference seen around 10 h post-infection (hpi) (34). However, no systematic study of SIE kinetics in the orthoflaviviruses could be found. Although the mechanism of interference in the orthoflavivirus system is not well understood, it is also thought to involve RNA replication components and limited cellular co-factors; in other words, both virus and host participate in the establishment of SIE (33, 53).

Previous studies of SIE in SINV infections have shown that SINV interferes with different alphaviruses in *Ae. albopictus* cells C6/36, C7-10, and U4.4 by several orders of magnitude, depending on the virus strain. The time points previously published were from the 48 h post-infection period (42). We took time points at 24, 48, and 72 hpi to allow the comparison of alphavirus SIE kinetics to that of orthoflavivirus kinetics. We did not see the same level of interference in our SINV control as previously published (45) for two possible reasons. In the report cited above, the acute infections were done at an MOI = 100 pfu/mL, and the resulting virus was titered on chick embryo fibroblast (CEF) secondary cultures, while ours were done at MOI = 10 and titered on C6/36 cells. Also, we previously demonstrated that SINV interfered with Aura, Semliki Forest, and Ross River viruses, showing heterologous interference. SINV infection did not interfere with a secondary infection of yellow fever virus, which illustrates a lack of SIE heterologous interference in a related arbovirus (orthoflavivirus). Although this early publication reported that SINV did not support heterologous SIE of an orthoflavivirus, our data have shown that SINV can interfere with DENV 4 in C6/36 cells, demonstrating a heterologous interference system.

**Fig 1:**
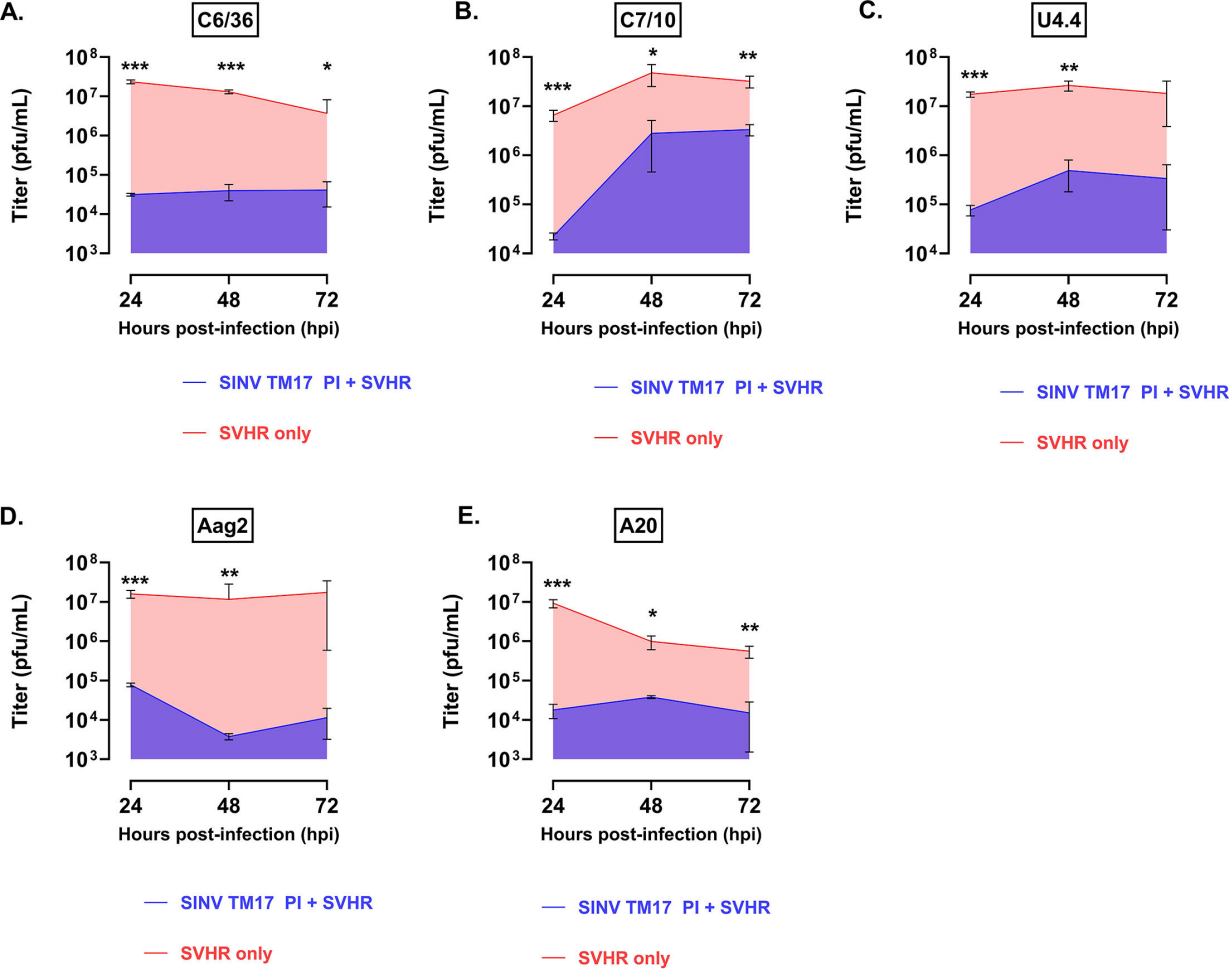
SIE results for *Ae. Albopictus* cells PI with SINV TM17 and challenged with SINV at 24, 48, and 72 hpi. Data points represent % control. Bars show the SE. (**A**) Results from C6/36 cells (99.7% reduction). (**B**) Results from C7-10 cells (93.3% reduction). (**C**) Results from U4.4 cells (99.8% reduction). (**D**) 99.8% inhibition. (**E**) 93% inhibition. *P ≤ 0.05, **P ≤ 0.01, ***P ≤ 0.001.

A second caveat necessary to measure SIE correctly was to substitute the RT-qPCR measurements of RNA for quantitation of genomic equivalents. Initially, the viral load of select infections in these cell lines was determined by RT-qPCR, using established and validated RT-qPCR assays. However, we found that cells PI with SINV in a superinfected culture produce more WT RNA than is produced by a single acute infection, precluding direct comparison. This was determined by measuring the amount of HR-LAV RNA compared to the WT virus RNA (data not shown). To replicate the findings of Condreay (42), SINV was used as the challenge virus to *Ae. albopictus* cells PI with SINV TM17. As shown in Fig. 1, the amount of virus suppressed was comparable to the amount of WT virus collected at 24 hpi in that study, ~2–3 orders of magnitude. Additionally, a secondary infection of SINV reduced WT virus production by ~3 orders of magnitude in all cells tested in that study. This indicates that the SINV HR-LAV is capable of establishing SIE at comparable levels of exclusion as seen previously (42). Therefore, the conditions used in the present study for the establishment of SIE are suitable for the examination of the ability of the HR-LAV system to produce SIE. The areas showing the levels of virus collected during this 3-day period are comparable to the SIE values reported herein for SINV and discussed below. For PI infections with SINV TM17, the WT infection was reduced 99.7% in C6/36 (Fig. 1A), 93.3% in C7-10 (Fig. 1B), and 99.8% in U4.4 cells (Fig. 1 and 3C). From Aag2 cells, inhibition was 99.8% and (Fig. 1E) 93% from A20 cells. SVHR is the prototype of the alphaviruses and served together with the companion HR-LAV strain SVHR TM17 as the control persistent infection to establish the correct conditions for these experiments in a reference strain model.

The establishment of persistence with each of the arboviruses in the *in vitro* system was monitored by a modified FISH plaque assay (discussed above) developed in this lab. For the SVHR strain studied, each of the cell clones was superinfected with SINV TM17, a well-studied alphavirus HR-LAV; CHIKV HR-LAV or DENV 1-4 HR-LAV was also studied and quantified using the FISH assay. Clear patterns of virus interference can be observed for these tests in SINV, CHIKV, and the DENV 1–4. These experiments serve as pilot experiments to refine a method to mitigate the circulation of pathogenic arboviruses. The establishment of an HR-LAV-immunized mosquito population that persists by natural means is predicted to impede pathogenic virus expansion. This method of pathogen regulation is expected to abate the circulation of pathogenic arbovirus while maintaining the ecology of the mosquito vectors in wild populations under natural conditions.

**Fig 2:**
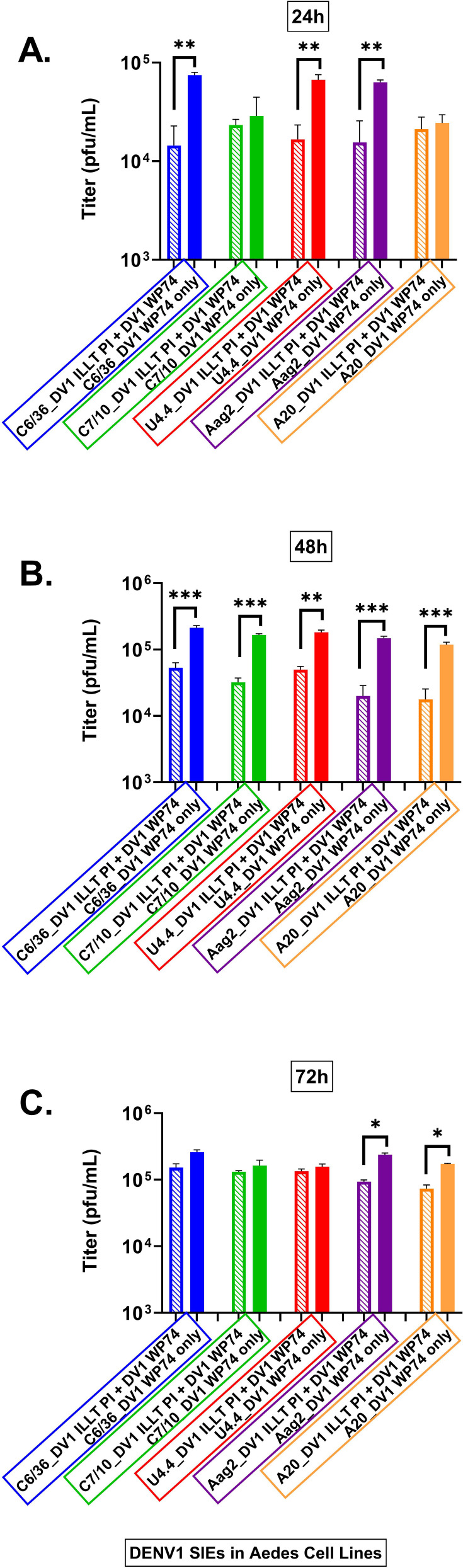
Levels of SIE in *Ae. albopictus* and *Ae. aegypti* mosquito cell lines infected with DV-1 ∆ILLT HR-LAV and challenged with WT DENV 1 WP74 and titered by plaque assay. (**A**) At 24 hpi, significant levels of DENV-1 were suppressed in C6/36, U4.4, and Aag2 cells. (**B**) At 48 hpi, all cell lines demonstrated significant SIE induced by HR-LAV DV1 ∆ILLT and challenged by DENV 1 WP74. (**C**) Measured at 72 hpi, significant SIE was restricted to the *Ae. aegypti* cells. Error bars signify SD. *P ≤ 0.05, **P ≤ 0.01, ***P ≤ 0.001.

As detailed in Materials and Methods, each of the *Aedes* spp. tested was infected with HR-LAV, and the infection was allowed to mature for 30 days into a PI culture. Each of the individual PI-infected cultures was then superinfected, and samples were taken at 24, 48, and 72 hpi. The experiment contained PI cells infected with CHIKV and DENV 1–4; each of the samples was collected and measured by FISH plaque assay. The result for DENV-1 is shown in Fig. 2. SIE in *Ae. albopictus* and *Ae. aegypti* mosquito cell lines infected with DV-1 ∆ILLT HR-LAV and challenged with WT DENV 1 WP74 sampled at 24, 48, and 72 hpi. At 24 hpi, significant levels of DENV-1 were suppressed in C6/36, U4.4, and Aag2 cells (Fig. 2A). All cells sampled at 48 hpi demonstrated significant SIE (Fig. 2B). When measured at 72 hpi, significant SIE was restricted to the *Ae. aegypti* cells (Fig. 2C).

**Fig 3:**
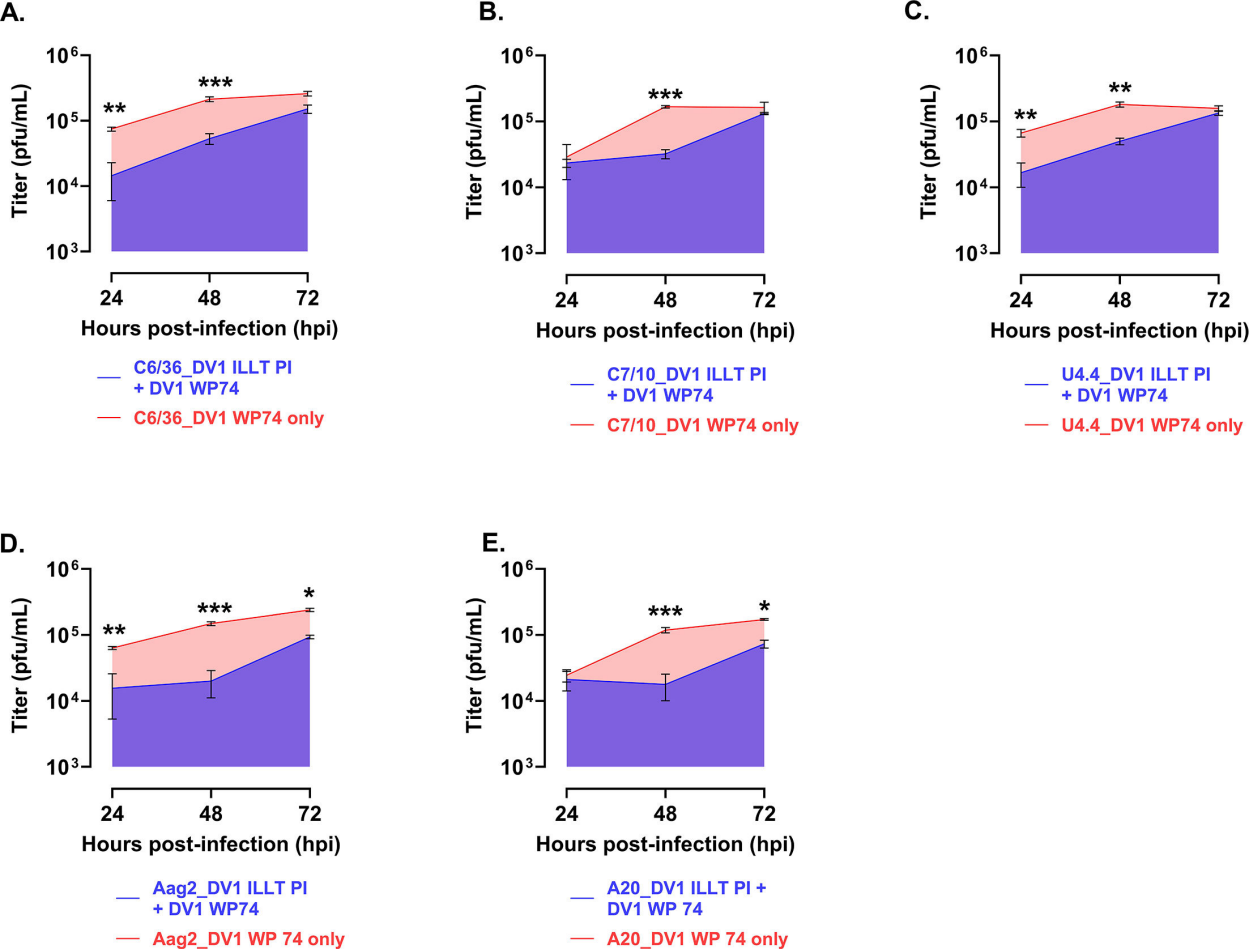
Shown are measurements of area under the curve for each of the cell lines tested for SIE with DV1 HR-LAV ∆ILLT challenged with DENV 1 WP’74 after 3 days of infection. All cell lines produced significant levels of interference compared to the control. (**A**) C6/36 SIE produced a 64% reduction. (**B**) C7-10 SIE produced a 58% reduction. (**C**) U4.4 produced 57%. (**D**) Aag2 produced 84% and (**E**) produced 70% interference. Error bars represent SE. *P ≤ 0.05, **P ≤ 0.01, ***P ≤ 0.001.

When DENV 1 SIE was measured as an area under the curve, the following levels of interference were determined. All cell lines produced significant levels of SIE. C6/36 cells produced 64% levels of interference, followed by C7-10 at 58%, U4,4 cells at 57%, Aag2 cells at 84%, and A20 cells at 70% all compared to the control infection. The charts are shown in Fig. 3.

**Fig 4:**
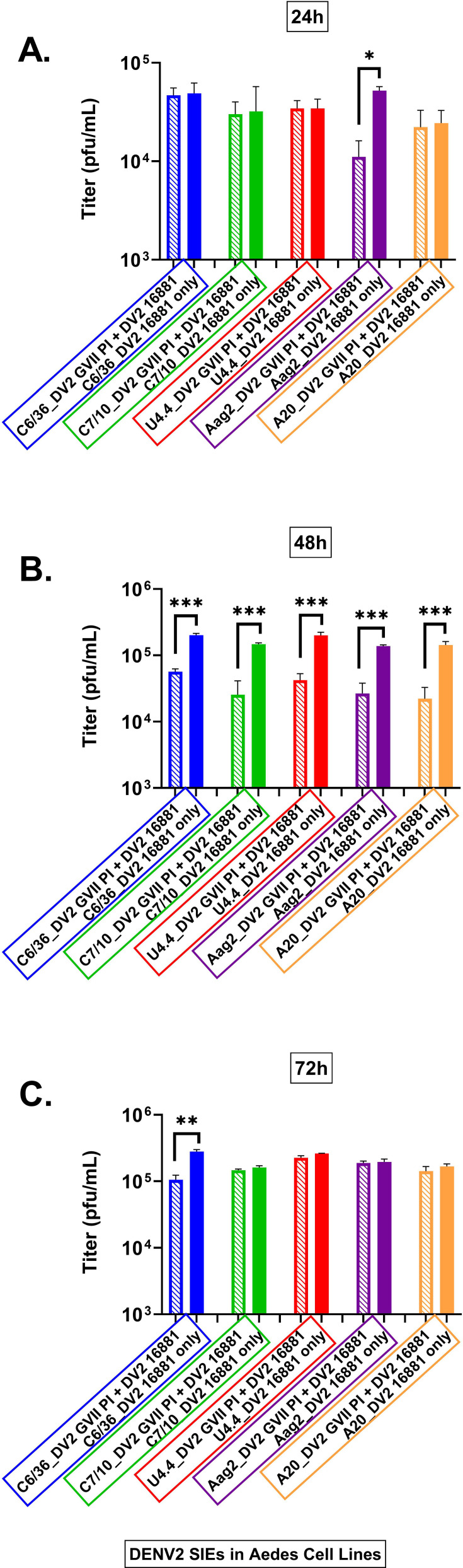
Levels of SIE in *Ae albopictus* and aegypti mosquito cells infected with DV-2 ∆GVII HR-LAV and challenged with DENV 2 16681 and titered by plaque assay. (**A**) At 24 hpi, significant levels of DENV-2 were suppressed in Aag2 cells. (**B**) At 48 hpi, all cell lines demonstrated significant SIE induced by HR-LAV DV2 ∆GVII and challenged by DENV 2 16681. (**C**) Measured at 72 hpi, significant SIE was restricted to the C6/36 cells. Error bars represent SD. *P ≤ 0.05, **P ≤ 0.01, ***P ≤ 0.001.

The results for the DV2 HR-LAV and DENV-2 16,684 WT virus are shown in Fig. 4. The levels of SIE in *Ae. albopictus* and *Ae. aegypti* mosquito cells infected with DV-2 ∆GVII HR-LAV and challenged with DENV 2 16681 are similar to those of DENV 1. At 24 hpi, significant levels of DENV-2 were suppressed in Aag2 cells (Fig. 4A). At 48 hpi, as with DENV 1, all cell lines demonstrated significant SIE induced by HR-LAV DV2 ∆GVII when challenged by DENV 2 16681 (Fig. 4B). At 72 hpi, significant SIE was restricted to the C6/36 cells (Fig. 4C).

**Fig 5:**
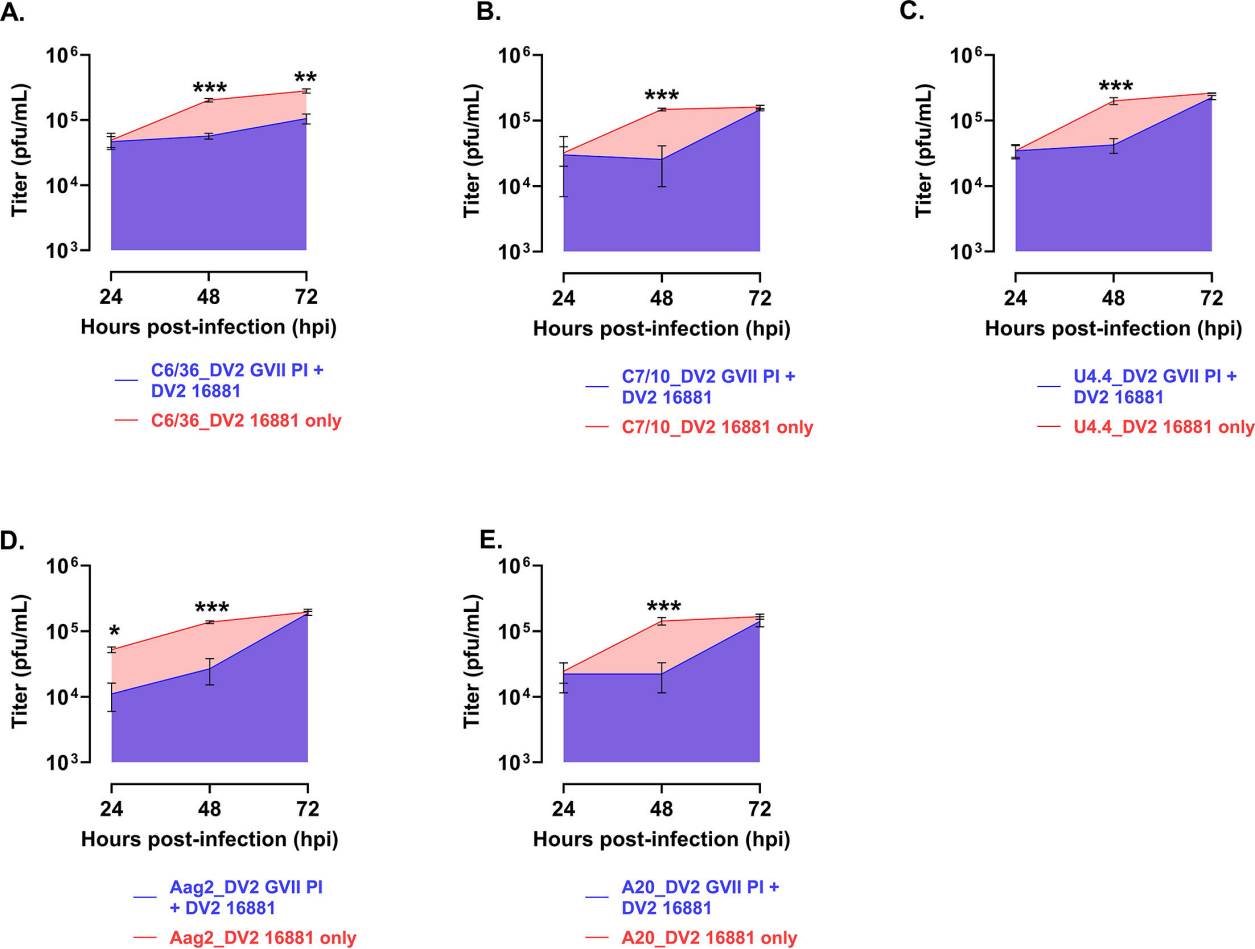
Shown are calculations of the area under the curve for each of the cell lines tested for SIE with DV2 HR-LAV ∆GVII challenged with DENV 2 16681 after 3 days of infection. (**A**) C6/36 cells produced 64% levels of interference. (**B**) C7-10 cells produced 58% interference. (**C**) U4.4 cells produced 57% interference. (**D**) Aag2 cells showed 84% interference. (**E**) A20 cells produced 70% interference. All measurements were compared to the control infection. Error bars represent the SD. *P ≤ 0.05, **P ≤ 0.01, ***P ≤ 0.001.

When DENV 2 SIE data were calculated, the following levels of interference were determined. All cell lines produced significant levels of SIE. C6/36 cells produced 64% levels of interference (Fig. 5A) followed by C7-10 58% (Fig. 5B), U4,4 cells 57% (Fig. 5C), Aag2 cells 84% (Fig. 5D), and A20 cells 70% (Fig. 5E) all compared to the control infection. The charts are shown in Fig. 5.

**Fig 6:**
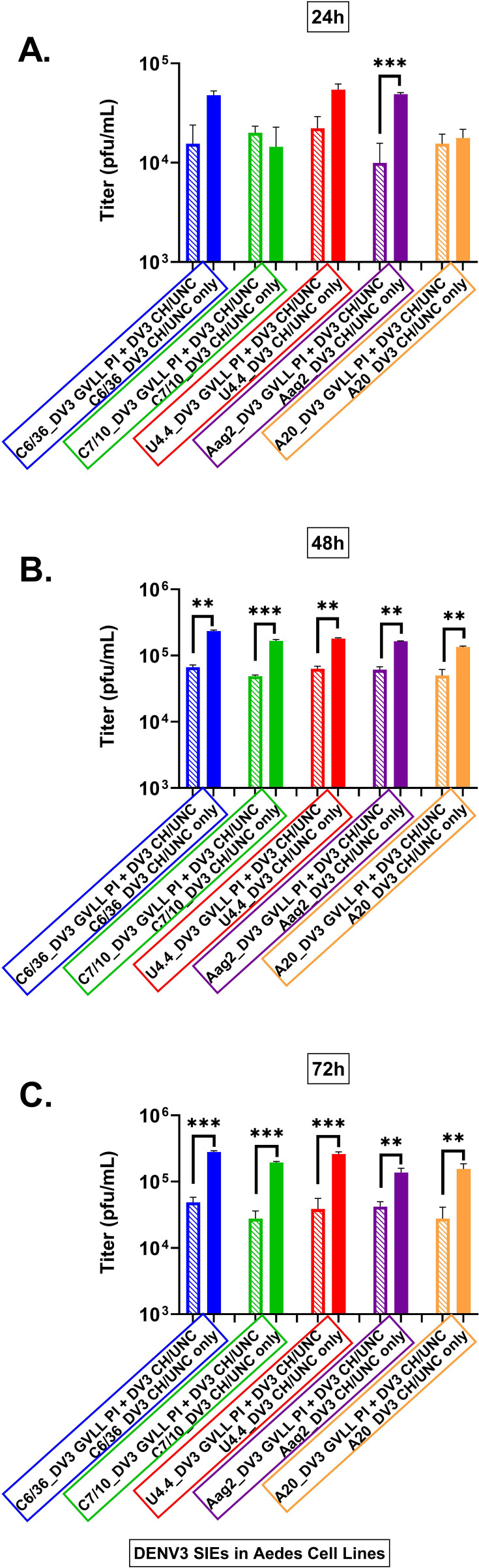
Shown are the levels of SIE in *Ae. albopictus* and *Ae. aegypti* mosquito cells infected with DV-3 ∆GVLL HR-LAV and challenged with DENV 3 CH/UNC. (**A**) At 24 h, Aag2 cells displayed a significant amount of SIE. (**B and C**) All cell lines tested showed a significant level of SIE at 48 and 72 hpi. Error bars represent SD. *P ≤ 0.05, **P ≤ 0.01, ***P ≤ 0.001.

The results for the DV-3 HR-LAV ∆GVLL and DENV-3 CH/UNC WT virus are shown in Fig. 6 and represent the data obtained for all the cells tested, sampled at 24, 48, and 72 h post-superinfection with the WT virus. At 24 hpi, a significant amount of SIE was detected from Aag2 cells (Fig. 6A). For this virus, all cells produced significant SIE at 48 (Fig. 6B) and 72 (Fig. 6C) hpi. This level of SIE was not seen for any of the other viruses tested.

**Fig 7:**
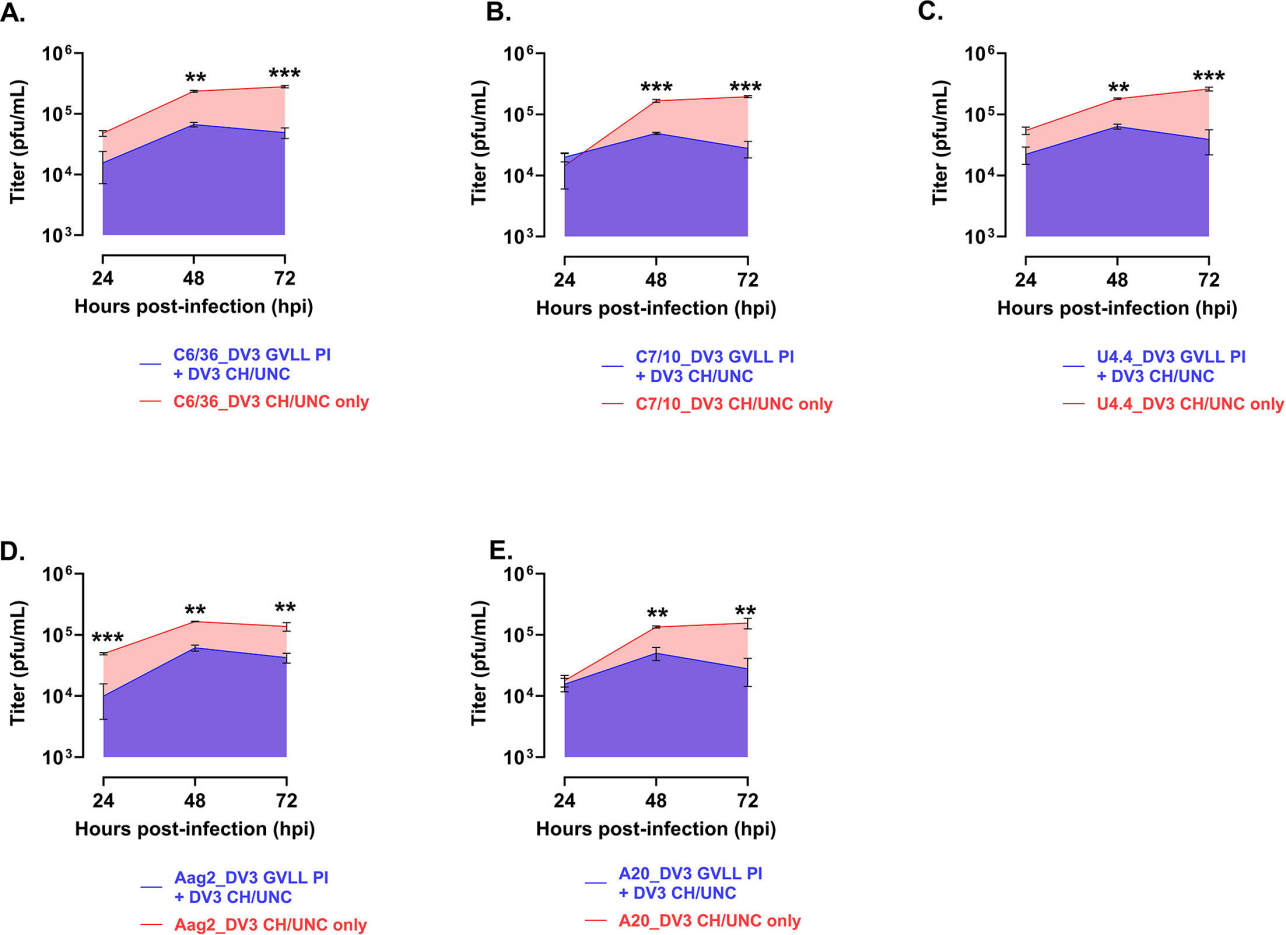
(**A**) Shown are calculations of the area under the curve for each of the cell lines tested for SIE with DV3 HR-LAV ∆GVLL challenged with DENV 3 CH/UNC after 3 days of infection. (**A**) In C6/36, 75% interference was observed. (**B**) In C7-10, 73% was observed. (**C**) In U4.4, 73% interference was observed. (**D**) In Aag2, 66% interference was observed. (**E**) In A20, 68% interference was observed. All interference was measured as a percentage of the WT control. Error bars represent SD. *P ≤ 0.05, **P ≤ 0.01, ***P ≤ 0.001.

When DENV 3 SIE was calculated as the area under the curve, the following levels of interference were determined. All cell lines produced significant levels of SIE, shown in Fig. 7. In C6/36, 75% interference was observed (Fig. 7A). Interference was observed in C7-10 (73%; Fig. 7B), U4.4 (73%; Fig. 7C), and Aag2 (66%; Fig. 7D). In Fig. 7E, A20, 68% interference was observed. All interference was measured as a percentage of the WT control.

**Fig 8:**
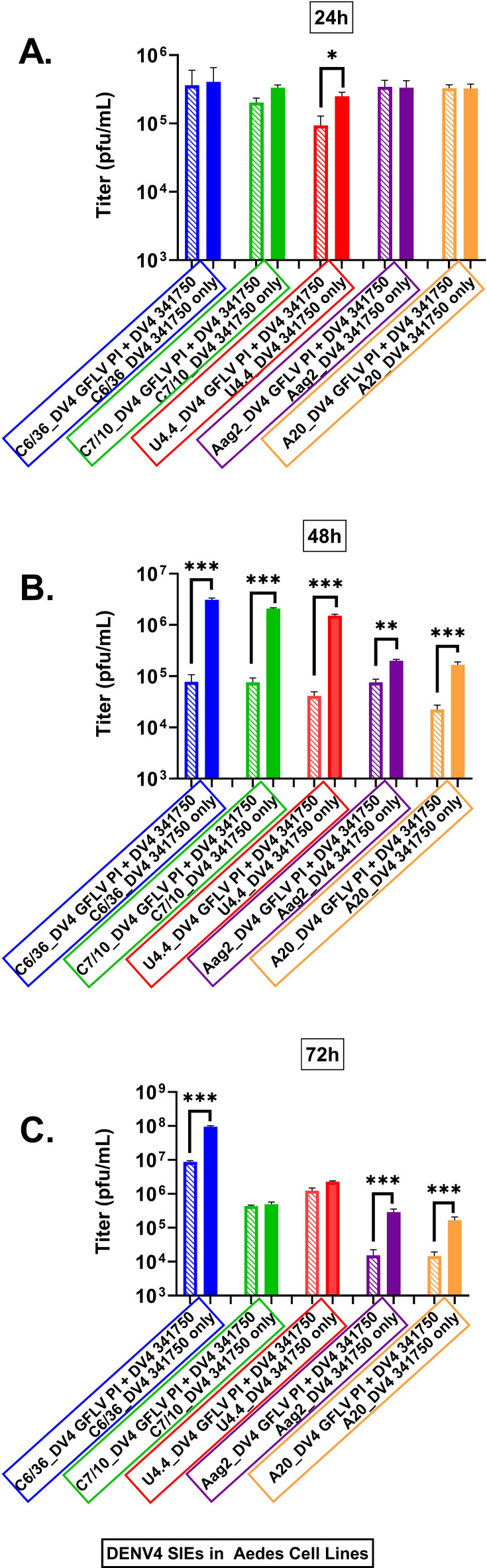
Shown are the levels of SIE in *Ae. albopictus* and *Ae. aegypti* mosquito cells infected with DV4 ∆GFLV HR-LAV and challenged with DENV 4341750. (**A**) Only U4.4 cells show SIE at 24 hpi. (**B**) As in all other DENV strains, the 48 hpi time point shows significant SIE for all cells tested. (**C**) At the 72 h time point, C6/36 cells, Aag2, and A20 cells displayed significant SIE. Error bars correspond to SE. *P ≤ 0.05, **P ≤ 0.01, ***P ≤ 0.001.

The final DENV tested was DENV 4 HR-LAV ∆GFLV. The experimental protocol was as detailed above. As shown in Fig. 8, the cell line that produced significant SIE at 24 h was U4.4 (Fig. 8A). Again, as with all the previous DENV strains, the 48 h time point produced the most significant time PI, which interfered with WT virus production (Fig. 8B). At 72 hpi, the virus continued to interfere in C6/36, Aag3, and A20 cells to different levels (Fig. 8C).

**Fig 9:**
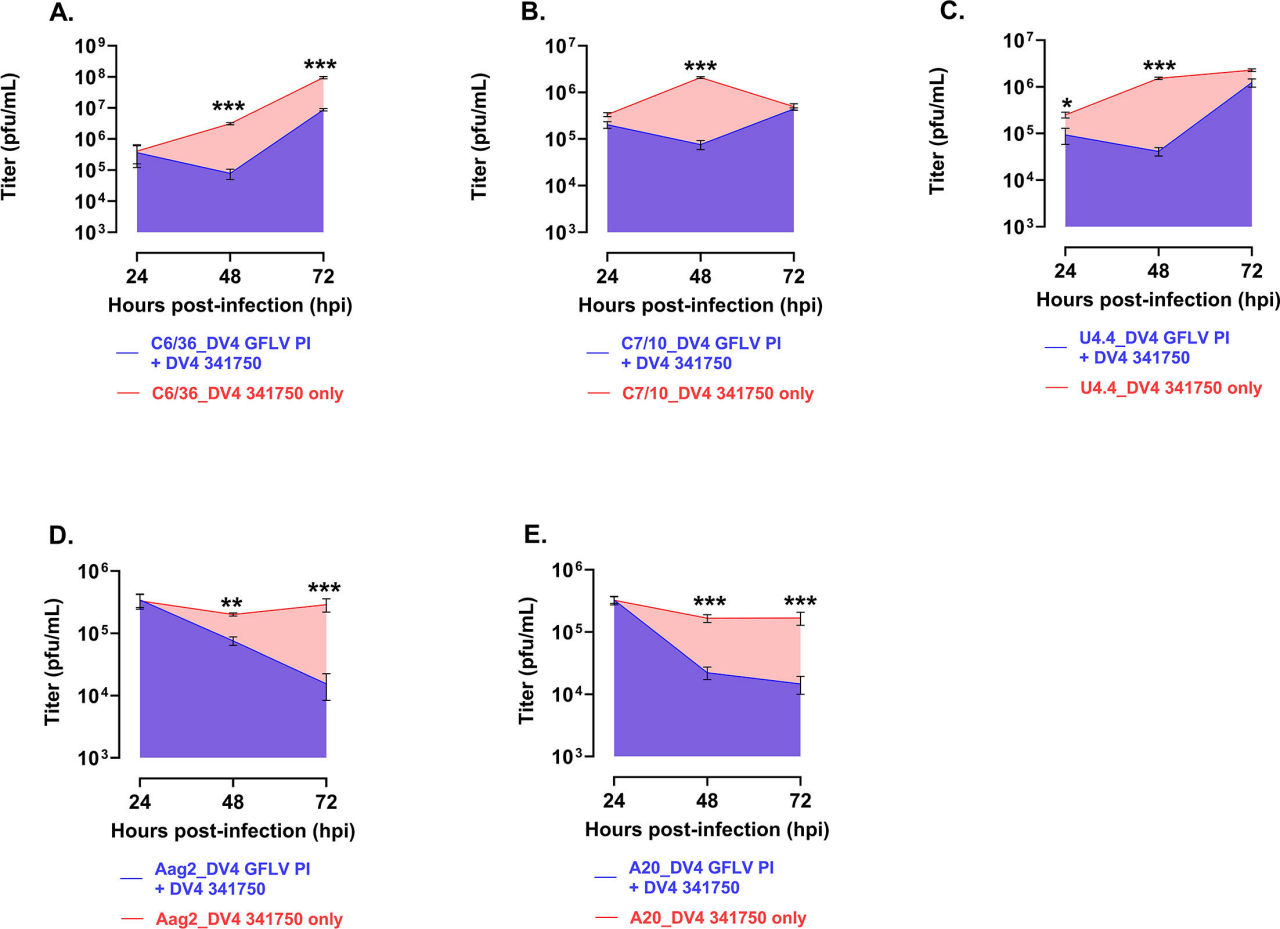
(**A**) Shown are measurements of area under the curve for each of the cell lines tested for SIE with DV3 HR-LAV ∆GVLL challenged with DENV 3 CH/UNC after 3 days of infection. (**A**) 91% for C6/36 cells, (**B**) 84% for C7-10 cells, (**C**) 75% for U4.4 cells, (**D**) 50% for Aag2 cells, and (**E**) 53% for A20 cells. Error bars correspond to SD. HR-LAV challenged with CHIKV 181/25 virus, titered by plaque assay. (**A**) All cell lines show significant SIE at 24 hpi. (**B**) At 48 hpi, C6/36 displays the highest titer, and as with DENV, this time point consistently produces significant levels of challenge virus suppression. In all other DENV strains, the 48 hpi time point shows significant SIE for all cells tested. (**C**) At the 72 h time point, C6/36 cells produce a large amount of virus and a significant titer. All other cells C7-10, U4.4, Aag2, and A20 cells displayed significant SIE. Error bars correspond to SE. *P ≤ 0.05, **P ≤ 0.01, ***P ≤ 0.001.

When the area under the curve is computed, the amount of interference is 91% for C6/36 cells (Fig. 9A), 84% for C7-10 cells (Fig. 9B), 75% for U4.4 cells (Fig. 9C), 50% for Aag2 cells (Fig. 8D), and 53% for A20 cells (Fig. 9E).

**Fig 10:**
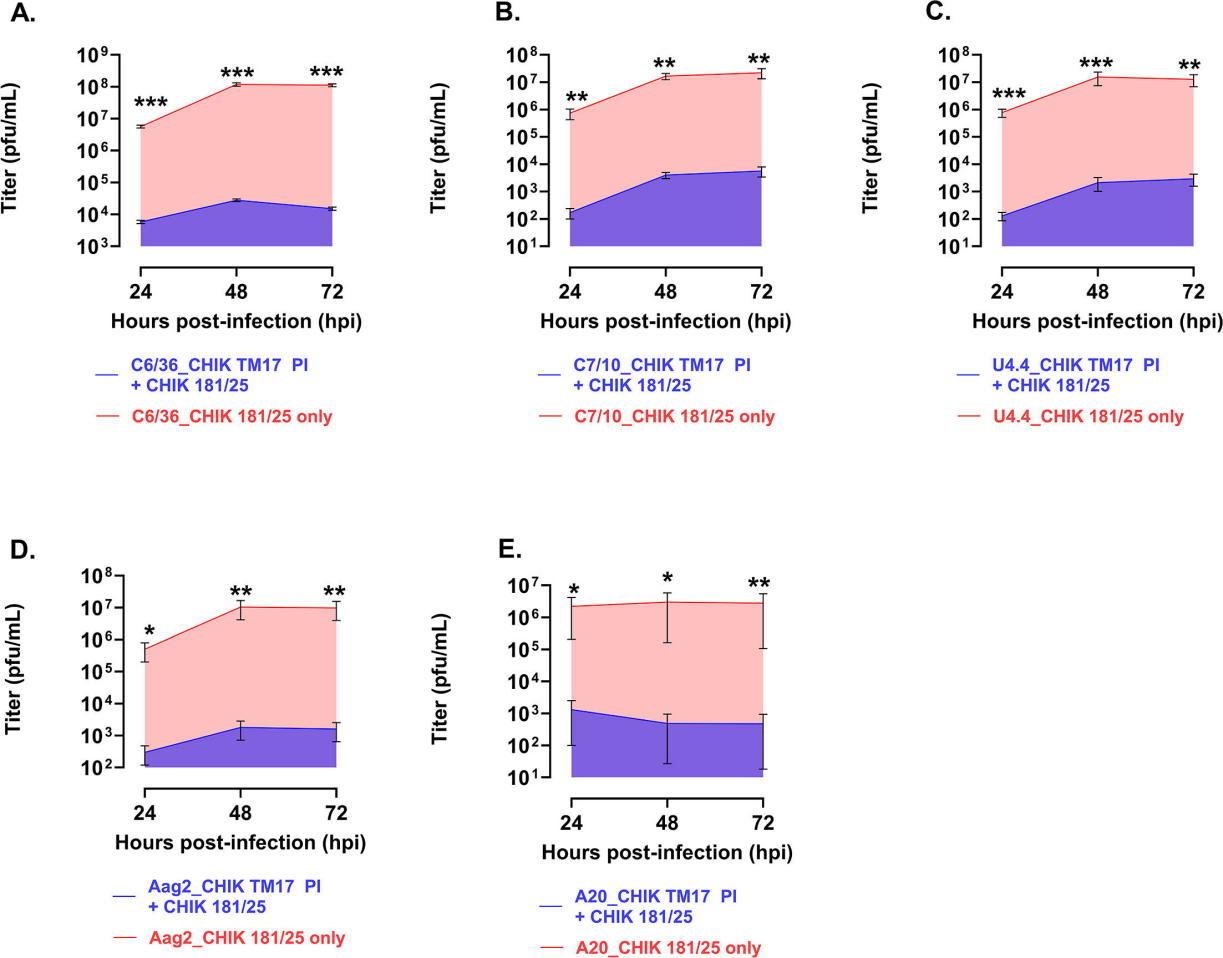
(**A**) Shown are measurements of area under the curve for each of the cell lines tested for SIE with CHIK TM17, challenged with CHIKV 181/25, and harvested 24, 48, and 72 hpi. (**A**) 99% for C6/36 cells, (**B**) 99% for C7-10 cells, (**C**) 99% for U4.4 cells, (**D**) 99% for Aag2 cells, and (**E**) 99% for A20 cells. All data points for these SIE infections were found to be significant. Error bars correspond to SD. *P ≤ 0.05, **P ≤ 0.01, ***P ≤ 0.001.

Also tested for the establishment of SIE was the HR vaccine strain of CHIKV, CHIK TM17, in a superinfection of the CHIK 181/25 strain used as WT. The same five *Aedes* spp. clones were made PI with HR CHIK TM17, superinfected with CHIKV 181/25, and media harvested at 24, 48, and 72 hpi, as with the dengue samples. CHIKV was found to be a superior suppressor of the second infection, giving 99% suppression in all cell lines tested up to 72 hpi. Results are shown in Fig. 10.

## DISCUSSION

**Fig 11:**
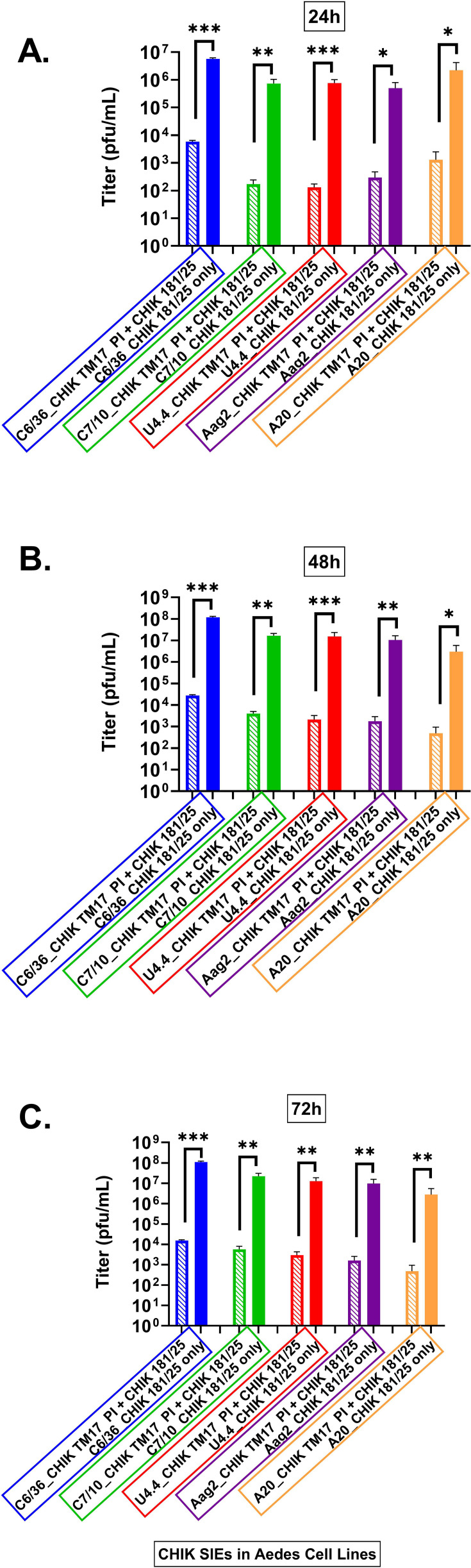
Shown are the levels of SIE in *Ae. albopictus* and *Ae. aegypti* mosquito cells infected with CHIK TM17 LAV and challenged with CHIKV 181/25 virus, titered by plaque assay. (**A**) All cell lines show significant SIE at 24 hpi. (**B**) At 48 hpi, C6/36 displays the highest titer, and as with DENV, this time point is consistently producing significant levels of challenge virus suppression. In all other DENV strains, the 48 hpi time point shows significant SIE for all cells tested. (**C**) At the 72 h time point, C6/36 cells are producing a large amount of virus and a significant titer. All other cells C7-10, U4.4, Aag2, and A20 cells displayed significant SIE. Error bars correspond to SE. *P ≤ 0.05, **P ≤ 0.01, ***P ≤ 0.001.

Our superinfection exclusion experiments in cultured mosquito cells are a systematic study of the amount of interference produced in five mosquito cell lines from two mosquito species *Ae. aegypti* and *Ae. albopictus*. The date presented above (summarized in Fig. 11) shows that a variety of insect cell lines can be infected with both alpha and flaviviruses. These results further show that SIE can be demonstrated when these cells are secondarily infected with wild-type homologous virus, with one very notable exception. When cells infected with the vaccine strain of dengue 2 are subsequently superinfected with wild-type dengue, suppression of wild-type replication is initially demonstrated; however, wild-type virus recovers and by 72 h grows to normal levels. We have no explanation for this result except to say that this is not the only instance where dengue 2 has shown its own peculiarities. We have made vaccines for all four serotypes of dengue. When the dengue 2 vaccine was injected into African Green Monkeys, it produced a strong immune response with large amounts of neutralizing antibody. It resulted in strong protection upon challenge. When the dengue 2 vaccine was injected in the tetravalent context (all vaccines present), it also produced a strong immune response with high levels of neutralizing antibody; however, when challenged with wild type, it did not protect. Again, this result cannot be explained but suggests that dengue 2 may have properties not shared by other flaviviruses.

The mechanism by which alphaviruses mediate SIE is well understood. In the normal course of infection, the RNA-dependent RNA polymerase (RDRP) initially copies the incoming plus-strand RNA to the negative strand to allow the production of progeny plus strands. At some point in the first 15–30 min of infection, a protease modifies the RDRP so that the production of negative strand is no longer possible. A superinfecting plus-strand RNA encounters this protease as it is translated, and the RDRP is one capable only of producing plus strands (48, 54; reviewed in reference 42). The mechanism by which orthoflaviviruses produce SEI is poorly understood but appears to be the result of a competition for resources where the initially infecting virus has the advantage (32; reviewed in reference 42).

These data also support the notion that SIE may provide a mechanism for control of these viruses in nature. In that regard, it is essential that these experiments be repeated on whole insects. We are currently seeking a collaboration to do that.
